# Accelerated brain aging in methamphetamine use disorder revealed by functional connectivity

**DOI:** 10.1093/nsr/nwag139

**Published:** 2026-03-05

**Authors:** Xinwen Wen, Jiahao Zhao, Wenhan Yang, Zhe Du, Dongcheng Wang, Suping Cai, Hongxian Shen, Dahua Yu, Jun Liu, Tifei Yuan, Kai Yuan

**Affiliations:** School of Life Science and Technology, Xidian University, China; School of Life Science and Technology, Xidian University, China; The Second Xiangya Hospital, Central South University, China; School of Life Science and Technology, Xidian University, China; School of Life Science and Technology, Xidian University, China; School of Life Science and Technology, Xidian University, China; The Second Xiangya Hospital, Central South University, China; School of Automation and Electrical Engineering, Inner Mongolia University of Science and Technology, Baotou, China; The Second Xiangya Hospital, Central South University, China; Shanghai Key Laboratory of Psychotic Disorders, Brain Health Institute, National Center for Mental Disorders, Shanghai Mental Health Center, Shanghai Jiao Tong University School of Medicine and School of Psychology, China; School of Life Science and Technology, Xidian University, China; School of Automation and Electrical Engineering, Inner Mongolia University of Science and Technology, Baotou, China; Xi’an Key Laboratory of Intelligent Sensing and Regulation of trans-Scale Life Information, School of Life Science and Technology, Xidian University, China; Engineering Research Center of Molecular and Neuro Imaging Ministry of Education, China

**Keywords:** methamphetamine-use disorder, normative model, brain age, functional connectivity, cognitive impairment

Drug addiction causes significant changes in behavior and body physiology [1], including cognitive function declines [2]. Epidemiological evidence indicates that substance use disorders are associated with increased dementia risk, as well as brain-volume reductions [3]. Such changes resemble the aged brain from both structural and functional aspects, yet there is currently a lack of empirical measurements linking addiction to brain aging.

Recently, the large-sample-size-based ‘brain-age model’ approach emerged [4], which may permit accurate estimation of the predicted age difference (PAD) [5] in individual brains. We first established three normalized brain-age prediction models based on 1076 healthy subjects (aged 18–81 years). Ethics approval was granted by the research ethics boards of the Second Xiangya Hospital/Central South University and all participants provided written informed consent. Resting-state functional connectivity (FC) patterns across 400 regions of interest (ROIs) [6] were extracted. Due to the high-dimensional nature of the data and the large sample size, we chose Lasso regression, gradient boosting regression and Gaussian process regression as the candidate models. We trained three brain-age models to fit the relationships between whole-brain connectivity features and chronological age in healthy controls (HCs) [7] (Fig. 1a). All three models achieved satisfactory performance in the training set (Pearson’s *r* = 0.9391, false discovery rate (FDR) *P* < 0.0001, 95% confidence interval (CI) = 0.9316–0.9458, mean absolute error (MAE) = 3.9458 for Lasso regression; Pearson’s *r* = 0.9964, FDR *P* < 0.0001, 95% CI = 0.9960–0.9968, MAE = 0.8735 for Gaussian regression; Pearson’s *r* = 0.9849, FDR *P* < 0.0001, 95% CI = 0.9830–0.9866; MAE = 1.8778 for gradient boosting regression). We then tested the reliability of the models in an independent set of HCs (*n* = 109). The results showed that the brain age predicted by the three models closely matched the individuals’ chronological age (Pearson’s *r* = 0.7672, 0.6586, 0.7276, MAE = 4.0968, 4.4979, 3.5202, respectively). Overall, our brain-age models provided an accurate and reliable estimation of an individual’s brain age (Fig. 1c).

**Figure 1.**
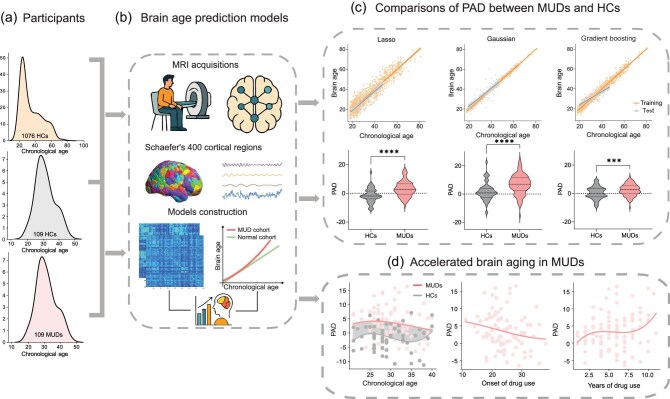
Methamphetamine addiction facilitates brain aging. (a) Participants in the current study. (b) Resting-state functional magnetic resonance imaging data were collected for the calculation of the resting-state functional connectivity matrices among 400 ROIs. Brain-age prediction models using Lasso regression, gradient boosting Regression and Gaussian regression were then constructed. (c) Performances of the three brain-age models in training and test datasets (top) and corresponding comparisons of the PAD between people with MUDs and HCs (bottom). (d) Accelerated brain aging in people with MUDs described by the changes in the PAD with chronological age as well as drug-use history (onset of drug use and years of drug use); the PAD curve was fitted by using polynomial estimation.

Next, we assessed PAD differences between 109 individuals with methamphetamine use disorder (MUD) and 109 age- and gender-matched HCs. The robustness of the findings was evaluated based on consistency across the three different models. We examined changes in the PAD with chronological age and drug-use history (including onset and duration of drug use) by fitting the PAD curve using polynomial estimation [8]. Accelerated brain aging in MUDs was further quantified by computing the ratio of the area under the curve (AUC) between people with MUDs and HCs. Comparisons revealed pronounced group differences in the age-related PAD, with people with MUDs exhibiting significantly higher PAD values across all three brain-age models compared with HCs (Cohen’s *d* = 0.8908, *P* < 0.0001 for Lasso regression; Cohen’s *d* = 0.7624, *P* < 0.0001 for Gaussian regression; Cohen’s *d* = 0.5011, *P* = 0.0003 for gradient boosting regression). We further examined associations between the PAD derived from Lasso regression (the largest effect size among the three models) and the clinical characteristics of people with MUDs (Fig. 1d). Specifically, the PAD–age trajectories indicated that the group difference was largest in early to mid-adulthood, and young people with MUDs exhibit the most pronounced brain-age acceleration, consistently with the neurodevelopmental vulnerability in late adolescence and young adulthood and early methamphetamine use impairing neuroplasticity. AUC refers to the area under the group-wise PAD–age trajectory. The ratio of the AUC of the PAD curve with the chronological age between the people with MUDs and HCs was 1.7, indicating a markedly greater degree of brain aging in people with MUDs. Additionally, higher PAD values in people with MUDs were associated with earlier onset of drug use and longer duration of drug use. We also used multiple linear regression with onset and years of drug use as predictors to examine their linear associations with the PAD. The regression equation was as follows:


(1) \begin{eqnarray*}
{\mathrm{PAD}} &&= - 0.2672{\mathrm{ }} \times {\mathrm{\ Onset\ of\ drug\ use }}\\
&&\,+ {\mathrm{\ }}0.1097{\mathrm{ }} \times {\mathrm{\ Years\ of\ drug\ use\ }}\\
&&+ {\mathrm{\ }}9.2335.
\end{eqnarray*}

Additionally, we examined the relationship between accelerated brain aging in people with MUDs and cognitive performance indexed by using the Digit Symbol Substitution Test (DSST)—a sensitive measure of processing speed and attention. Across the three brain-age models, the PAD values in people with MUDs were negatively correlated with the DSST scores (Lasso regression: Pearson’s *r* = −0.5733, *P* = 0.0053; Gaussian regression: Pearson’s *r* = −0.4265, *P* = 0.0477; gradient boosting regression: Pearson’s *r* = −0.4428, *P* = 0.0390). Consistently with this association, people with MUDs also showed lower DSST scores than age- and gender-matched HCs (*t* = 2.807, *P* = 0.0068). Such accelerated brain aging is connected to measurable cognitive impairment.

These findings provide robust evidence that methamphetamine addiction robustly accelerates brain aging. Individuals with earlier onset of drug use and longer duration of use exhibited exacerbated brain aging. Notably, brain aging is connected to declined cognitive functions. The brain-aging clock may serve as a potential biomarker for disease severity, rehabilitation assessment and interventions for drug addiction.

There are several limitations to our study. The effect of education (closely related to cognitive reserve) and socioeconomic conditions on brain aging in people with MUDs remain unclear. Brain-aging trajectories and the functional connectome may differ by sex [9], suggesting that sex-related differences may partially confound the estimates of brain-age acceleration in MUD. We included sex as a covariate at several steps in the Supplemental file and found that brain-age acceleration in the MUD group was statistically significant, indicating that our findings are not entirely driven by sex imbalance. However, the sex imbalance in our sample may have reduced the statistical power to detect sex-specific effects and limited the generalizability of the findings, underscoring the need for sex-stratified brain-age prediction models in future research. Importantly, it is necessary to investigate the mechanism underlying the observed acceleration and the generalizability of our findings to other substance use disorders by using multimodal neuroimaging [10]. Finally, the specificity of brain aging on MUD vs. prolonged methamphetamine use (e.g. through prescription) need to be explored in the future.

## Supplementary Material

nwag139_Supplemental_Files
