# Supplementary material for: Accelerated brain aging in methamphetamine use disorder revealed by functional connectivity
Source: Natl Sci Rev. 2026 Mar 5;13(8):nwag139. doi: 10.1093/nsr/nwag139 (PMC13134444; doi:10.1093/nsr/nwag139)
Supplement: nwag139_Supplemental_Files [file nwag139_supplemental_files.zip › Supplemental_file_marked.docx]

**Supplementary Materials and Methods**

**Participants**

We recruited 1,294 participants (624 female) aged 18–81 years (mean = 33.99, SD = 12.57). The training set consisted of 1,076 healthy individuals, while the independent validation set included 109 healthy controls (Table 1). Diagnoses were established with structured clinical interview according to DSM-5 criteria. Participants with use disorders for alcohol, cannabis, cocaine, opioids, sedatives, or other substances were excluded. Occasional alcohol/tobacco use without a DSM-5 diagnosis was permitted. All sites excluded individuals with: current or past DSM-5 substance use disorders (including stimulant use disorders); major psychiatric disorders; neurological conditions; current psychoactive medication use; MRI contraindications. A total of 109 age- and gender-matched MUD inpatients were recruited from drug rehabilitation centers. Inclusion criteria included a primary diagnosis of MUD with a duration of at least one year. Participants had only MUD as their primary addiction diagnosis. Exclusion criteria were current or prior psychiatric or medical disorders, or use of any medications within the preceding three months. All participant completed their clinical interview with psychiatrists. Resting-state functional MRI (fMRI) data were acquired across multiple sites using 3T MRI scanners (General Electric [GE], Philips, and Siemens; Table 2). The training set was derived from data collected at sites 1-11, while the testing cohort comprised data from sites 1,2, and 11. Data from MUDs were exclusively collected at site 11.

**MRI processing and Calculation of PAD**

*Data Processing*

Resting-state functional MRI (fMRI) data were preprocessed using the Data Processing & Analysis for Brain Imaging toolbox (DPABI_V7.0, <http://rfmri.org/dpabi>) including slice timing, realignment, spatial normalization, Gaussian smoothing, and nuisance signal regression (white matter, cerebrospinal fluid (CSF), global signal). The preprocessed data was subsequently used for calculation of the resting state functional connectivity matrices among 400 ROIs using Pearson correlation and Fisher-Z transformation.

*Eliminating site differences*

The participants were recruited from multiple research sites, which may compromise the comparability of functional connectivity features. To correct the functional connectivity matrices, the ComBat algorithm was implemented using Python. Specifically, the site information for each participant was extracted and used as batch label. The ComBat algorithm was applied through the process of estimating batch effect parameters, adjusting the feature matrix, and reconstructing features free from batch-related bias. Finally, the corrected functional connectivity features were yielded for subsequent analysis.

*Building brain-age prediction models*

We constructed brain age prediction models using Lasso regression, GradientBoosting Regression, and Gaussian Regression. The performances of the brain age models were evaluated on an independent validation set, using Pearson’s correlation coefficient and mean absolute error (MAE). Subsequently, we recorded each participant’s initial predicted brain age and the original prediction bias, performed age bias correction, and constructed an error correction model to fit the distribution pattern of systematic errors. This step yields the predicted systematic error for each participant, allowing the calculation of the corrected predicted brain age and the predicted age difference (PAD). This metric has eliminated systematic error and reflects the true individual differences in brain aging relative to the population average.

*Sex distribution and potential impact*

Sex differences in brain aging trajectories have been reported in prior literature, and an imbalanced sex distribution could potentially influence brain-age estimates and between-group comparisons. We note that sex-related differences in functional connectome and age-related trajectories may lead to systematic offsets in brain-age prediction. To address this concern, we used gender as a covariate when applying the ComBat algorithm to correct batch effects in the functional connectivity matrices; and in the regression model, we captured both linear and nonlinear effects of actual age and gender on brain age while excluding compounded confounding due to gender factor; brain age was predicted from model residuals after removing confounding effects, including gender.

**Statistical analysis**

The performance of the brain age models was evaluated using Pearson’s correlation coefficient and mean absolute error (MAE) between predicted and chronological ages. Group differences in PAD were assessed using two-sample t-tests (two-tailed, p < 0.05). All statistical results were corrected for multiple comparisons using the false discovery rate (FDR) method (p < 0.05). We examined dynamic changes in PAD with chronological age and drug use history (including onset and duration of drug use) by fitting the PAD curve via polynomial estimation. Accelerated brain aging in MUDs was further quantified by computing the area under the curve (AUC) ratio between MUDs and HCs. AUC refers to the area under the group-wise PAD-age trajectory, representing the cumulative excess aging of the group across the entire age span. Additionally, we used Pearson correlation analysis to examine the relationship between accelerated brain aging in MUDs and cognitive performance indexed by the Digit Symbol Substitution Test (DSST), a sensitive measure of processing speed and attention. The differences of DSST scores between MUDs and age- as well as HCs were tested using two-sample t-tests.

**Table 1. Demographic and clinical characteristics of the participants**

| **Group** | **Training set** | **Validation set** | **MUD cohort** |  |  |
| --- | --- | --- | --- | --- | --- |
|  | **Healthy individuals**  **(N=1076)** | **Healthy controls**  **(N=109)** | **MUDs**  **(N=109)** | **Test** | **p value** |
| **Age(years)** | 34.56±13.44 | 31.21±6.07 | 31.21±6.07 | F=7.810 | P=0.0004 |
| **Gender(M/F)** | 468/608 | 101/8 | 101/8 | χ²=175.49 | P<0.0001 |
| **Education(years)** | N/A | N/A | 9.99±3.05 | N/A | N/A |
| **Age of first use(years)** | N/A | N/A | 26.07±6.33 | N/A | N/A |
| **Craving** | N/A | N/A | 4.35±2.15 | N/A | N/A |
| **Drug use(years)** | N/A | N/A | 5.63±2.43 | N/A | N/A |

**Table 2. MRI Scanner Specifications and Acquisition Parameters Across Sites**

| Center | Scanner | Number | TR | TE | FOV | Matrix | Resolution | Slices | Thickness | Gap | Volume |
| --- | --- | --- | --- | --- | --- | --- | --- | --- | --- | --- | --- |
|  |  |  | (ms) | (ms) | (mm^2^) |  | (mm^2^) |  | (mm) | (mm) |  |
| Site1 | GE HDxT 3T | 253 | 2000 | 40 | 240×240 | 64×64 | 3.75×3.75 | 35 | 3 | 0 | 200 |
| Site2 | GE HDxT 3T | 120 | 2000 | 30 | 220×220 | 64×64 | 3.44×3.44 | 33 | 4 | .6 | 180 |
| Site3 | GE HDxT 3T | 34 | 2000 | 30 | 220×220 | 64×64 | 3.44×3.44 | 36 | 3 | 1 | 185 |
| Site4 | GE HDxT 3T | 66 | 2000 | 30 | 240×240 | 64×64 | 3.75×3.75 | 33 | 4 | 0 | 250 |
| Site5 | PHILIPS Achieva 3T | 46 | 2200 | 35 | 230×230 | 128×128 | 1.80×1.80 | 50 | 3 | 0 | 240 |
| Site6 | Siemens Trio 3T | 73 | 2000 | 30 | 210×210 | 64×64 | 3.28×3.28 | 30 | 4 | .8 | 210 |
| Site7 | GE EXCITE 3T | 35 | 2000 | 30 | 220×220 | 64×64 | 3.44×3.44 | 30 | 5 | 0 | 200 |
| Site8 | Siemens Trio 3T | 254 | 2000 | 30 | 220×220 | 64×64 | 3.44×3.44 | 32 | 3 | 1 | 242 |
| Site9 | Siemens Trio 3T | 109 | 2500 | 27 | 220×220 | 64×64 | 3.44×3.44 | 43 | 3.4 | 0 | 200 |
| Site10 | GE MR750 3T | 82 | 2000 | 40 | 220×220 | 64×64 | 3.44×3.44 | 32 | 4 | .5 | 180 |
| Site11 | Magnetom Skyra 3T | 222 | 2000 | 30 | 220×220 | 64×64 | 3.44×3.44 | 36 | 4 | 0 | 225 |
