# Supplementary material for: Accelerated brain aging in methamphetamine use disorder revealed by functional connectivity
Source: Natl Sci Rev. 2026 Mar 5;13(8):nwag139. doi: 10.1093/nsr/nwag139 (PMC13134444; doi:10.1093/nsr/nwag139)
Supplement: nwag139_Supplemental_Files [file nwag139_supplemental_files.zip › Supplementary Table.docx]

**Table 1. Demographic and clinical characteristics of the participants**

| **Group** | **Training set** | **Validation set** | **MUD cohort** |  |  |
| --- | --- | --- | --- | --- | --- |
|  | **Healthy individuals**  **(N=1076)** | **Healthy controls**  **(N=109)** | **MUDs**  **(N=109)** | **Test** | **p value** |
| **Age(years)** | 34.56±13.44 | 31.21±6.07 | 31.21±6.07 | F=7.810 | P=0.0004 |
| **Gender(M/F)** | 468/608 | 101/8 | 101/8 | χ²=175.49 | P<0.0001 |
| **Education(years)** | N/A | N/A | 9.99±3.05 | N/A | N/A |
| **Age of first use(years)** | N/A | N/A | 26.07±6.33 | N/A | N/A |
| **Craving** | N/A | N/A | 4.35±2.15 | N/A | N/A |
| **Drug use(years)** | N/A | N/A | 5.63±2.43 | N/A | N/A |

**Table 2. MRI Scanner Specifications and Acquisition Parameters Across Sites**

| Center | Scanner | Number | TR | TE | FOV | Matrix | Resolution | Slices | Thickness | Gap | Volume |
| --- | --- | --- | --- | --- | --- | --- | --- | --- | --- | --- | --- |
|  |  |  | (ms) | (ms) | (mm^2^) |  | (mm^2^) |  | (mm) | (mm) |  |
| Site1 | GE HDxT 3T | 253 | 2000 | 40 | 240×240 | 64×64 | 3.75×3.75 | 35 | 3 | 0 | 200 |
| Site2 | GE HDxT 3T | 120 | 2000 | 30 | 220×220 | 64×64 | 3.44×3.44 | 33 | 4 | .6 | 180 |
| Site3 | GE HDxT 3T | 34 | 2000 | 30 | 220×220 | 64×64 | 3.44×3.44 | 36 | 3 | 1 | 185 |
| Site4 | GE HDxT 3T | 66 | 2000 | 30 | 240×240 | 64×64 | 3.75×3.75 | 33 | 4 | 0 | 250 |
| Site5 | PHILIPS Achieva 3T | 46 | 2200 | 35 | 230×230 | 128×128 | 1.80×1.80 | 50 | 3 | 0 | 240 |
| Site6 | Siemens Trio 3T | 73 | 2000 | 30 | 210×210 | 64×64 | 3.28×3.28 | 30 | 4 | .8 | 210 |
| Site7 | GE EXCITE 3T | 35 | 2000 | 30 | 220×220 | 64×64 | 3.44×3.44 | 30 | 5 | 0 | 200 |
| Site8 | Siemens Trio 3T | 254 | 2000 | 30 | 220×220 | 64×64 | 3.44×3.44 | 32 | 3 | 1 | 242 |
| Site9 | Siemens Trio 3T | 109 | 2500 | 27 | 220×220 | 64×64 | 3.44×3.44 | 43 | 3.4 | 0 | 200 |
| Site10 | GE MR750 3T | 82 | 2000 | 40 | 220×220 | 64×64 | 3.44×3.44 | 32 | 4 | .5 | 180 |
| Site11 | Magnetom Skyra 3T | 222 | 2000 | 30 | 220×220 | 64×64 | 3.44×3.44 | 36 | 4 | 0 | 225 |
